# Obituary Notices

**Published:** 1847-06

**Authors:** 


ARTICLE XI.

OBITUARY.

Died, suddenly, on the 9th of May, Dr. George McClellan of Philadelphia, in the 51st year of his age.

Dr. McClellan stood confessedly in the first rank of surgeons, not only in this country, but of the present time. He was bold, skillful, fertile in resources, inventive and original in a high degree. We have not at hand the materials to enable us to give, at this moment, an examination of his most brilliant achievements in surgery. His operations and improvements are nowhere collected together and recorded, but scattered through the pages of many different periodicals and books, and disseminated and spread with a liberal hand among his pupils and the profession at large. We may say, however, that scarcely a difficult operation can be named which he had not performed many times, and in some instances improved upon the mode of their performance.

He was a man of extensive knowledge out of his own profession; of warm and generous sympathies; of a kind and liberal heart. We are much pleased to notice the respect paid to his memory in Philadelphia, which has, in his death, sustained severe loss. We have not heard whether the intention he long entertained of recording his experience in a systematic form, as a treatise on surgery, had been carried into effect, but if not he has left behind him no monument worthy of his mind and talents.

On the 10th of May, of consumption, Cicero Robbe, M. D., of Pleasant Grove, Illinois.

Dr. Robbe was a graduate of the Medical College at Chicago, and during the short time he had been in practice (about one year,) had gained an extensive reputation, and gave promise of eminence. He was expected to give the course on anatomy at the summer term of Rush Medical College, but was prevented by ill health.

On the 29th of April last, of pneumonia, John Revere, M. D., Professor of Theory and Practice of Medicine in the University of New York, in the 60th year of his age.

Professor Revere was an accomplished and popular teacher. He lived highly respected, and died deeply regretted.

On the 2d of April last, Hon. John Cotton, M. D., of Marietta, Ohio.

He was an ornament to the profession, by his great learning, sound judgment, and solid piety.

On the 5th of May last, A. L. Warner, M. D., Professor of Surgery in Hampden Sydney College, Virginia.

